# Availability of essential medicines and pharmaceutical inventory management practice at health centers of Adama town, Ethiopia

**DOI:** 10.1186/s12913-019-4087-0

**Published:** 2019-04-25

**Authors:** Adane Teshome Kefale, Hafiza Hayredin Shebo

**Affiliations:** 1 Department of Pharmacy, College of Health Sciences, Mizan-Tepi University, Mizan Aman, Ethiopia; 2 College of Health and Medical Sciences, School of Pharmacy, Haramaya University, Harar, Ethiopia

**Keywords:** Essential medicines, Tracer drugs, Stock management

## Abstract

**Background:**

Good inventory management practices in the health facilities are one of the critical aspects that influence the availability of essential medicines (EMs). This study aimed to assess EMs availability and inventory management practices at health centers (HCs) of Adama town, Ethiopia.

**Method:**

Institution based cross sectional survey was conducted among six HCs in Adama Town from March 19 to April 12, 2017. Self-administered questionnaire and observational checklists were used to collect quantitative information. Eleven tracer drugs (TDs) that were selected by the Federal ministry of health and included in the TD list of the HCs were used to assess the availability of EMs at the time of the survey; and during the past 12 months. The data were entered and analyzed using SPSS version 21. The accuracy of record keeping was assessed using inventory management assessment tool (IMAT) indicators.

**Result:**

Five HCs had Essential drug list and the procurement was made as per the list. Out of six HCs, four of them procured EMs from both pharmaceutical fund and supply agency (PFSA) of Ethiopia and private suppliers. Stock status of PFSA and transportation were the major challenges during the procurement process. The overall average availability of TDs on the day of the survey was 76.3%. Average length of stock out days for TDs during the past 12 months from each HC was 40.6 days. Among the TDs assessed at HCs, oral rehydrating salt was stock outed for 144 days while paracetamol was stock outed only for 1.4 days. The discrepancy of TDs between physical count and on bincard for which physical inventory less than the balance on bincard ranges from 0 to 33.3%.

**Conclusion:**

The availability of EMs was low and there was also poor inventory management practice in some of the HCs during the study period.

## Background

Medicines are vital components of patient care all over the world. World Health Organization (WHO) define essential medicines (EMs) as products that satisfy the priority healthcare needs of the population which should be available in the health facities at all times in adequate amounts with affordable price by the community [1].

Ethiopia has a national drug policy aimed to ensure adequate supply of medicines which are required for treatment of diseases affecting the majority of the country’s population [2], which gives the primary mandate to the government. To achieve this, the country developed national list of EMs which guides the decision of all health service providers with regard to selecting and availing the most needed medicines at every level of the healthcare system at all times with affordable cost [3].

Inventory management is vital for pharmaceutical supply system which involves the management of routine pharmaceutical ordering process. It helps to maintain a steady supply to patients, hence preventing product stock out, while minimizing the costs of holding inventory [4]. Accurate and updated stock records are crucial for proper inventory management since they are input to calculate future needs. Holding stocks is important to ensure availability of essential items almost all the time. The selection of items to stock should rely on their value to public health and volume of consumption [4, 5]. Nowadays, inventory management is assisted with computerized Logistics Management Information System (LMIS) that allows easy recording of all medicines transactions and connects all levels of supply chains [6, 7]. Despite the importance of LMIS in improving efficiency of the overall inventory management process [8, 9], it is underutilized in developing countries like Ethiopia [10].

Inventory management plays a major role in providing efficient healthcare in relation to three vital aspects of drug supplies used in the health facilities; availability, safety, and affordability. Those are the most important determinants of quality of care and patient satisfaction with services provided in public health facilities [11, 12]. Poor inventory management might lead to overstocking or under stocking of EMs which subsequently results wastage of resource and increased morbidity and mortality due to shortage of life saving drugs [13–17].

Ministry of health of Ethiopia developed standardized inventory management tools expected to be utilized by public health facilities throughout the country whether operated manually or use LMIS. These are bin card, stock card, Internal Facility Request and Resupply form (IFRR), Report and Requisition Form (RRF). The bin card is kept with the product inside the store and up dated in every transactions while stock card is similar to the bin card but is used to track stock based on issuing and receiving orders. The IFRR voucher is used to report internal transfer of items between the facility’s pharmaceutical store and dispensing units. The RRF is used to order health commodities from PFSA whereas RRUC is used to track the transfer of supplies back to PFSA. The ministry recommends physical inventory and updating of bin cards and stock cards on regular basis [6].

As per the WHO recommendation, availability of EMs should be 100%, but it is estimated that about one-third of the world population does not have access to medicines, particularly in Africa and Asia [18, 19]. Availability of generic medicines at public sectors is less than 60% across WHO regions, especially in Africa region [20].

In Ethiopia, EMs stock out is still a common problem although the degree is variable from facility to facility, ranged from 26 to 91% [7, 21–28]. In addition, poor inventory management challenges availability of EMs in the country [7, 22, 28]. Thus, it is crucial to assess the scope of problem in availability of EMs and inventory management practice of HCs in Adama town for interventions.

## Methods

### Study area, design and sampling technique

The study was conducted at HCs in Adama Town, East Shewa of Oromia region, Ethiopia; located 100 km East of the capital, Addis Ababa. The town has seven functional HCs namely; Geda HC, Dembela HC, Adama HC, Hawas HC, Biftu HC, Anole HC and Boku HC. Institution based cross sectional study was conducted at six HCs from March 19 to April 12, 2017. The availability of EMs was assessed at the date of survey and the previous 12 months, from March 2016 to March 2017.

The inclusion criteria of the study were presence of functional store and dispensary unit, EMs and health professionals working at the site for at least 6 months, while medical supplies, equipment and reagents were excluded from the study.

Out of seven HCs in Adama town, six were included in this study. Eleven trace drugs (TDs), which are representatives of EMs expected to be found in HCs, were used to assess availability of EMs at the day of survey and during the previous 12 months. The TDs used for this study were Coartem, rifampicin/isoniazid/ethambutol/pyrazinamide fixed dose combination, Medroxy progesterone injection, Pentavalent vaccine, Amoxicillin 500 mg capsule, Oral Rehydrated Salt (ORS), Mebendazole 100 mg tablet, Tetracycline eye ointment 1%, Paracetamol 500 mg tablet, ergometrine injection and Ferrous Salt plus Folic acid.

The outcome variables of the study were availability of EMs and pharmaceutical inventory practice of HCs in the study area. Data were collected on explanatory variables including sociodemographics of health professionals, use of LMIS, source of drug supply, availability of EDL, functionality of DTC, the presence of guideline for selection, forecasting and procurement, lead time required for supply of procured drugs, transportation during procurement process, the use of FEFO/FIFO and type of logistic forms/records used to manage EMs

### Data collection and analysis

A structured questionnaire adapted from Logistics Indicator Assessment Tool (LIAT) was used to collect data. LIAT is a tool developed by the USAID funded DELIVER which is used to conduct institution-based survey to assess health commodity logistics system performance and commodity availability at health facilities [29]. The questionnaire contains information about availability of EDs, use of LMIS, methods of inventory management and procurement process. Availability of TDs was also assessed using observational checklist to review data from bin cards. Accuracy of record keeping was evaluated by performing physical count of TDs to estimate the discrepancy between the recorded value and the counted value.

Data was collected by two trained druggists. Data quality was assured by pretesting at Mizan HC and training of data collectors on objectives of the study and techniques of data collection. The principal investigators continuously supervised the collection and daily check consistency of the collected data.

The collected quantitative data was entered and analyzed using SPSS 21.0 version for windows. The descriptive data was summarized using frequency and average. For each HC, three indicators were assessed to evaluate stock management practices using the Inventory Management Assessment Tool (IMAT) developed by INFORM program at Management Science for Health [30]. The tool is used to assess the effectiveness of record-keeping and stock management practices in a health facility and provides suggestions for improvement. The indicators used are percentage of recorded balances that is less than physical counts, percentage of recorded balances that is greater than physical counts, and average percentage of days the products were out of stock.

## Results

### General characteristics of the study setting

Of the seven HCs in Adama town, six (*n* = 6) HCs were included in this study. One of the HC was excluded due to lack of enough records to assess inventory management practice of EMs.

The data collected using questionnaire included six HCs responses whereas the data from the checklists includes response of five HCs. The data was collected both from pharmacy professionals at the dispensary unit and from store keepers of each HCs. Out of six HCs, only two HCs have two personnel (one pharmacist and one druggist) at dispensary units, while the others had only one personnel either pharmacist or druggist at dispensary units.

Among six HCs, five had pharmacists whereas one had druggist at dispensary units. The type of health professionals as a store keeper was different for each HC, only two had pharmacists as a store keeper, three had druggist while one HC store was managed by nurse.

### Availability of essential medicines

Availability of EMs was assessed by interviewing store keepers on most frequently stock out medicines and observation with checklist. On average, 76.4% of TDs were available at the day of visit in each HC. The result indicate that on average, each HC was stock outed 41.8% of TDs during the past 12 months (Table 1). Of the 11 TDs, six were available in all HCs at the day of visit while ergrometrine injection was stock outed at day of visit and in the last 12 months in all HCs. Interview of stoke keepers revealed that ORS, Amoxacillin syrup 125 mg/5 ml, TTEO, FEFOL tab and Ergometrine injection were the most frequently stock outed drugs.

**Table 1:** Availability of EMs during day of visit and stock out review over 12 months of TDs (*n* = 11) at five HCs in Adama Town, March, 2017

Health Centers	Availability at survey period N (%)	Stock out during survey period N (%)	Stock out past, 12 months N (%)
Anole	10 (90.9%)	1 (9.1%)	2 (18.2%)
Biftu	7 (63.6%)	4 (36.4%)	5 (45.5%)
Dembela	8 (72.7%)	3 (27.3%)	3 (27.3%)
Gedda	10 (90.9%)	1 (9.1%)	5 (45.5%)
Adama	7 (63.6%)	4 (36.4%)	8 (72.7%)
Average	8.4 (76.4%)	2.6 (23.6%)	4.6 (41.8%)

**Fig. 1:**
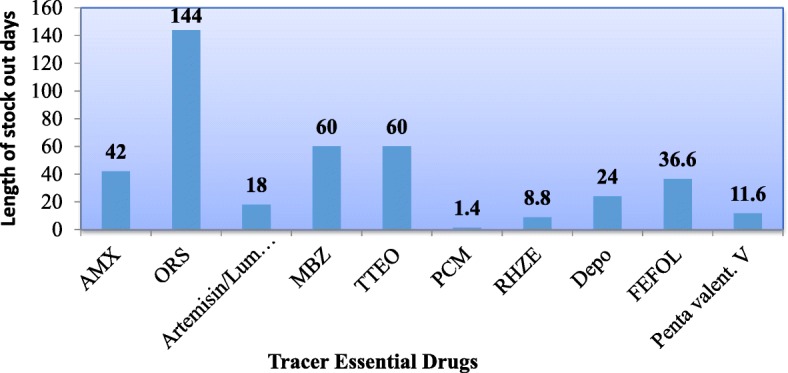
Average length of stock out days for TDs within the past 12 months at five HCs, Adama town, 2017. (AMX: amoxicillin, MBZ: mebendazole, TTEO: tetracycline eye ointment, PCM: paracetamol, RHZE: Rifampicin, Isoniazid, Pyrazinamide, Ethambutol, Depo: medroxy progesterone, FEFOL: ferrous salt plus folic acid, Pentanta valent. V: pentavalent DPT-HEP-Hib Vaccine)

Excluding ergometrine injection, the average stock out days of TDs from all five HCs within the past 12 months was 40.6 days. Among five HCs, ORS was stock out for longer period of time (144 days) whereas paracetamol was stock out for shorter duration which was 1.4 days (Fig. 1).

### Inventory management practice of the health centers

**Fig. 2:**
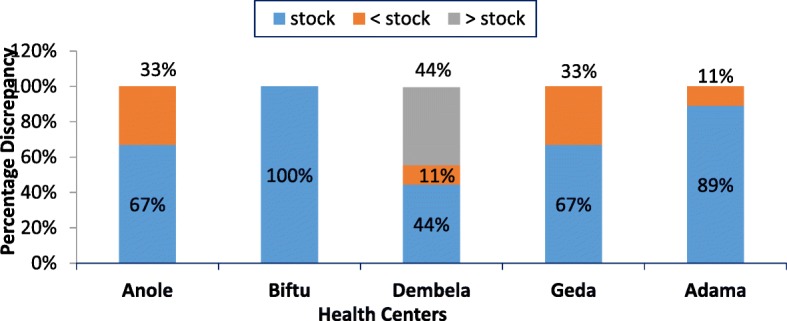
Percentage discrepancy between physical count and balance on bin card of TDs at five HCs of Adama Town, 2017

The accuracy of record keeping system in the HCs is summarized by three indicators. Accurate stock record balance ranges from 44.4 to 100% while percentage of stock record balance that were less than physical inventory ranges from 0 to 33.3%, whereas, percentage of stock record balance that were greater than physical inventory ranges from 0 to 44.4%. The physical inventory was greater than the recorded balance only for one HC (Fig. 2).

All HCs (six) determined their own drug supply using formula based on their annual consumption. Detail of inventory management practice of HCs is depicted in Table 2.

**Table 2:** Inventory management practices of HCs, Adama Town, 2017. (*n* = 6)

Variables		Adama HC	Anole HC	Biftu HC	Dembela HC	Geda HC	Hawas HC
Bin card available (updated)	Amoxicillin	Yes (Yes)	Yes (Yes)	Yes (Yes)	Yes (No)	Yes (Yes)	NA
	ORS	Yes (Yes)	Yes (Yes)	Yes (Yes)	Yes (Yes)	Yes (Yes)	NA
	Mebendazole	Yes (Yes)	Yes (Yes)	Yes (Yes)	Yes (No)	Yes (No)	NA
	TTEO	Yes (Yes)	Yes (Yes)	Yes (Yes)	Yes (Yes)	Yes (Yes)	NA
	Paracetamol	Yes (Yes)	Yes (Yes)	Yes (Yes)	Yes (No)	Yes (Yes)	NA
	FEFOL	Yes (Yes)	Yes (Yes)	Yes (Yes)	Yes (No)	Yes (No)	NA
	RHZE	Yes (Yes)	Yes (Yes)	Yes (Yes)	Yes (No)	Yes (Yes)	NA
	Depo provera	Yes (Yes)	Yes (Yes)	Yes (Yes)	Yes (Yes)	Yes (Yes)	NA
	Ergometrine	No (No)	No (No)	No (No)	No (No)	No (No)	NA
	Coartem	Yes (Yes)	Yes (Yes)	Yes (Yes)	Yes (Yes)	No (No)	NA
	Pentavalent V	No (No)	No (No)	No (No)	No (No)	No (No)	NA
Annual physical inventory		Yes	No	Yes	No	No	No
Policy and guideline for LMIS		Yes	Yes	No	Yes	Yes	No
Functional computer system		Yes	No	No	No	Yes	No
Logistic forms used	Bin cards	Yes	Yes	Yes	Yes	Yes	No
	Stock cards	No	Yes	No	Yes	No	No
	RRF	Yes	Yes	Yes	Yes	Yes	Yes
	IFRR	Yes	No	Yes	Yes	Yes	No
	Facility form	Yes	No	No	No	No	No
FEFO/FIFO drug distribution system in the facility		Yes	Yes	Yes	Yes	Yes	No (random)
Own EDL available		Yes	Yes	Yes	No	Yes	Yes
Functional DTC available		Yes	No	Yes	Yes	Yes	No
Who select drugs for EDL inclusion		DTC	Pharmacy unit only	DTC	DTC	By HC head	Pharmacy unit only
Source of drug	PFSA	Yes	Yes	Yes	Yes	Yes	Yes
	Private supplier	Yes	No	Yes	No	Yes	Yes
Transportation of drugs	From PFSA	The HC	Health bureau	Health bureau	Health bureau	The HC	PFSA
	From private suppliers	The HC	NA	Health bureau	NA	The HC	Heath bureau
Lead time	From PFSA	Less 1 week	1 to 2 month	Less 1 week	Less 1 week	Less 1 week	2 months
	From privates	Less 1 week	NA	Less 1 week	NA	Less 1 week	2 weeks to 1 month

## Discussion

The study was aimed to evaluate availability of EMs and pharmaceutical inventory management practices among HCs in Adama town. Availability of drugs largely influence quality of healthcare and patient satisfaction. Stock out of EMs enforces patients to use more costier alternatives. Drug availability is affected by efficiency of inventory management.

The overall availability of EMs in this study was far from WHO recommended target point [12]. In 2009, the general availability of EMs in Ethiopia was reported to be 91% but, study conducted at different part of Ethiopia after 2009 showed big variation on the availability of EMs ranged from 26 to 91% [22, 27]. The availability of TDs documented in this study is better than Jimma and Amhara region but, lower compared to Gondar and Addis Ababa [16–20]. The reason behind might be due to the stock status of regional PFSA and the budget put on primary public health facilities vary from one region to the other in Ethiopia. When the result of this study compared to Tanzania, it is lower but much higher compared with Ugandan study [15, 31]. Stock outing of EMs at the day of survery in this study (23.6%) is higher than previous studies in Tanzania (20%) [15] and Gondar, Ethiopia (9%) [22].

The severity of stock out duration of TDs in each HC was assessed by calculating the average stock out days of TDs within the past 12 months. The 1 year average stock out days of TDs in this study is lower when compared to the situation in Uganda, 72.9 days, and higher when compared to the result in Gondar, 30.5 days, [22, 31]. The reason for high stock out period in this study might be due to transportation during procurement process and unavailability of the TDs at PFSA as most professionals stated. Of the 11 TDs, ORS was stocked out for longer period when compared to the other TDs whereas, in Gondar from 26 TDs chloroquine syrup was stocked out for more days [22]. The long stock out period for ORS might be due its’ high consumption since it is important lifesaving medicine for diarrheal diseases, common in developing countries like Ethiopia. In addition procurement of drug for HCs is highly bureaucratic. The government allow purchase of drugs from private supplies only when unavailable in PFSA. It also limit quantity of drug to be purchased. High consumption, in the absence of fluidy supply system cause frequent stock out and cumulative longer day of stock out.

The physical count that is less than the balance on bin card is one of the indicators for poor record keeping practice. In this study, the percentage of discrepancy varies among all HCs, highest in Dembela HC. From the store keeper’s response, although there is guideline to use LMIS in this HC, the absence of computer software system and lack of training on logistic forms result poor record keeping practice in this HC. The discrepancy was high when compared to one of the public hospitals in Tanzania and lower than one of the HC in Gondar town [13, 22]. Ideally, a facility should not have discrepancies between the physical inventories and the balance on bin card for each item but, in practice there is acceptable level of errors. In general, discrepancies of more than 10% may require efforts to improve data quality [29]. Thus, except one HC all the other needs to improve their record keeping practice.

Utilization of different logistic forms is variable among HCs of this study. Bin cards were used in majority of HCs and updated on transaction which better than reported from Uganda primary health facilities [32]. Effective inventory management should be supported by LMIS. But in this study only two HCs in Adama town used LMIS in their facility which was low compared to the study done at HCs in Addis Ababa [7]. This might be due to lack of software system to manage their store and lack of training on LMIS in most HCs in Adama town.

Regarding lead time, this study revealed that the lead time was as long as 2 months when EMs procured from PFSA. It is longer when compared to the lead time reported from Addis Ababa, which was 1 week to 2 weeks [7]. This might be due to the stock status of regional PFSA varies from region to region.

This study is limited to one geographical area and single facility level (health centers) only due to financial constraints. Extrapolation should be taken with precaution with reviewing similar studies in other settings to get full picture of the country.

## Conclusion

The availability of EMs and the accuracy of record keeping in the HCs were low. The major problem common for all HCs in the procurement process were PFSA stock status and transportation. The absence of computer software system and lack of enough pharmacy professionals are some of the challenges to perform inventory management practice in the HCs properly, which further decrease the availability of EMs in the facility. We recommend health proffessionals working at respective health centers to improve inventory management practice. The regional health bureau should provide capacity building to HCs with provision of computers and trainings.

## Abbreviations

DTC: Drug and Therapeutic committee
EDL: Essential drug list
EMs: Essential medicines
FEFO: First expire first out
FIFO: First in first out
HCs: Health centers
IFRR: Internal facility request and resupply form
IMAT: Inventory management assessment tool
LMIS: Logistic management information system
PFSA: Pharmaceutical fund and supply agency
RRF: Report and requisition form
RRUC: Record for returning unusable commodities
TDs: Tracer drugs
WHO: World Health Organization
